# Metabolic Disorders and Inflammatory Bowel Diseases: Unraveling Shared Pathways and Clinical Interactions

**DOI:** 10.3390/metabo16030181

**Published:** 2026-03-09

**Authors:** Fotios Fousekis, Afroditi Lamprou, Maria Saridi, Ioanna Nefeli Mastorogianni, Konstantinos Mpakogiannis, Georgios D. Lianos, Konstantinos H. Katsanos

**Affiliations:** 1 Department of Gastroenterology, University Hospital of Ioannina, 45500 Ioannina, Greece; 2 Medical Department 2, Academic Teaching Hospital Fürth, Friedrich-Alexander Universität Erlangen-Nürnberg, 90766 Fürth, Germany; 3 Department of Nursing, University of Thessaly, 41500 Larissa, Greece; 4 Department of Surgery, University Hospital of Ioannina, 45500 Ioannina, Greece; glianos@uoi.gr

**Keywords:** inflammatory bowel disease, metabolic syndrome, gut microbiota, insulin resistance, adipose tissue

## Abstract

Inflammatory bowel diseases (IBDs) and metabolic disorders are increasingly recognized as interconnected conditions that frequently coexist and influence each other’s clinical course. Accumulating evidence indicates that patients with IBD face a substantial burden of obesity, metabolic syndrome, metabolic dysfunction-associated steatotic liver disease, osteoporosis, and type 2 diabetes. These associations appear to be driven by shared and interacting mechanisms, including intestinal barrier disruption, gut microbiota dysbiosis, chronic systemic inflammation, and adipose tissue-mediated immunometabolic pathways. Metabolic comorbidities may worsen IBD activity, reduce response to therapy, increase complications, and contribute to higher health care utilization. Conversely, intestinal inflammation and commonly used treatments, particularly corticosteroids, can adversely affect glucose metabolism, lipid metabolism, body composition and bone homeostasis. Advanced therapies have demonstrated variable metabolic effects, some of which may be beneficial through suppression of systemic inflammation. Recognition of these bidirectional interactions highlights the importance of routine metabolic screening and integrated, multidisciplinary management. Lifestyle interventions, nutritional optimization and individualized therapeutic strategies represent central parts of comprehensive management.

## 1. Introduction

Inflammatory bowel diseases (IBDs), comprising Crohn’s disease (CD) and ulcerative colitis (UC), are chronic immune-mediated disorders of the gastrointestinal tract characterized by relapsing intestinal inflammation and systemic immune activation [1]. The pathogenesis of IBD is complex and multifactorial, arising from the interplay between genetic susceptibility, environmental exposures, dysregulated immune responses and alterations in the gut microbiota. Aberrant activation of both innate and adaptive immune pathways plays a central role, driving persistent intestinal inflammation and progressive tissue damage [2,3]. Clinically, IBD manifests with a spectrum of gastrointestinal symptoms, including abdominal pain; chronic diarrhea, often bloody in UC; and unintended weight loss. In addition, IBD is frequently accompanied by extraintestinal manifestations, reflecting its systemic inflammatory nature [4,5].

The epidemiology of IBD has changed substantially over recent decades. IBD remains most prevalent in North America, Northern and Western Europe, and Australasia, where prevalence exceeds 0.3% of the population. The highest reported prevalence rates reach 505 per 100,000 for UC in Norway and 322 per 100,000 for CD in Germany, while in North America prevalence is approximately 286 and 319 per 100,000, respectively [6]. While incidence has stabilized in many high-income countries, rapidly increasing rates are now observed in newly industrialized regions across Asia, South America, and Africa, reflecting a global epidemiological transition [7]. IBD may affect all age groups, with a peak onset between 15 and 35 years and a smaller second peak in older adults, and its burden is rising among pediatric and elderly populations worldwide [8]. UC shows similar prevalence between sexes, whereas CD exhibits regional and age-related sex differences [9].

In parallel with the global rise in IBDs, the prevalence of metabolic disorders has increased markedly, raising important questions about shared pathogenic mechanisms and clinical interactions. Emerging data indicate a bidirectional association between IBDs and metabolic conditions [10,11]. Metabolic disorders comprise a broad group of diseases resulting from disturbances in biochemical metabolism, involving abnormalities in the processing of carbohydrates, lipids, proteins, or nucleic acids. These disturbances may arise from genetic defects, environmental factors, or their interaction, leading to impaired metabolic pathways and the accumulation or deficiency of specific metabolites [12,13]. Acquired metabolic disorders include diabetes mellitus, obesity, osteoporosis, dyslipidemia, metabolic dysfunction-associated steatotic liver disease (MASLD) and metabolic syndrome. These conditions typically develop later in life and are largely driven by environmental exposures, lifestyle factors, and chronic inflammation. Metabolic syndrome, in particular, is defined by a constellation of interrelated risk factors, including central obesity, dyslipidemia, hypertension, and hyperglycemia, which collectively increase the risk of cardiovascular disease and type 2 diabetes [14,15].

Metabolic disorders and IBDs appear to be linked through overlapping pathophysiological, genetic and microbiome-related mechanisms. Chronic, low-grade systemic inflammation observed in metabolic conditions such as obesity, type 2 diabetes mellitus, and metabolic syndrome has been associated with impaired intestinal barrier integrity and immune activation, which may contribute to increased susceptibility to IBD or modulation of disease activity [16]. Conversely, sustained intestinal inflammation in IBD may promote metabolic dysfunction, including insulin resistance and an increased cardiovascular risk, potentially mediated by persistent cytokine signalling and changes in adipokine profiles [17].

Given the growing prevalence of both conditions and their clinical interplay, a comprehensive understanding of these interactions is therefore needed. In this review, we aim to provide an updated synthesis of the epidemiological associations, pathophysiological pathways, and therapeutic interactions between IBDs and metabolic disorders. Beyond summarizing existing evidence, we provide an integrated overview of the bidirectional mechanisms linking IBDs and metabolic disorders, including differences in metabolic expression between disease subtypes. In addition, we examine the impact of metabolic comorbidities on IBD course, treatment response, and long-term outcomes. Finally, we discuss implications for systematic metabolic screening, risk assessment, and multidisciplinary management in routine clinical practice.

## 2. Review Type and Literature Search Strategy

This manuscript represents a narrative review based on a structured and comprehensive literature search. A systematic search of the PubMed/MEDLINE and Scopus databases was conducted to identify relevant studies published up to January 2026. The following search terms were used: (“metabolic syndrome” OR “obesity” OR “diabetes” OR “metabolic disorders” OR “osteoporosis” OR “metabolic dysfunction-associated steatotic liver disease”) AND (“inflammatory bowel disease” OR “ulcerative colitis” OR “Crohn’s disease”).

In addition, the reference lists of relevant articles were manually screened to identify further eligible studies. Abstracts were carefully reviewed, and full-text articles were assessed to select the most relevant publications for inclusion. Eligible studies were required to address epidemiological, pathophysiological, or therapeutic interactions between IBDs and metabolic disorders. Non-English articles and conference abstracts without full-text publication were excluded.

Review articles were used to contextualize the field and identify key primary studies. Human studies were prioritized to ensure clinical relevance, while animal and in vitro studies were included to clarify mechanistic pathways and highlight areas where evidence remains limited. For epidemiological associations, priority was given to large population-based studies and recent meta-analyses, mainly from the past five years, to ensure up-to-date pooled estimates and contemporary data. Formal risk-of-bias assessment was not performed due to the narrative nature of the review.

## 3. Epidemiological and Clinical Interactions Between Metabolic Disorders and IBDs

Epidemiological studies increasingly describe the coexistence of IBDs and metabolic comorbidities. The metabolic disorder subtypes with the highest prevalence in IBD patients include metabolic syndrome, MASLD and obesity. Metabolic syndrome and MASLD are reported at higher rates in IBD populations, with a more pronounced prevalence in UC patients than in CD patients [18] (Table 1 and Table 2).

### 3.1. Metabolic Syndrome

Metabolic syndrome is one of the most commonly reported metabolic conditions in IBD cohorts. According to a recent meta-analysis involving 2501 patients, the pooled prevalence of metabolic syndrome in individuals with IBD was 19.4%. The prevalence of metabolic syndrome was reported to be significantly higher in UC than in CD (38.2% vs. 13.6%, *p* = 0.03) [19]. This prevalence is comparable to or slightly lower than the prevalence in the general adult population, which recent global data estimate at 25–39% depending on region, age, and population characteristics [39,40]. In matched cohort studies, the prevalence of metabolic syndrome in IBD patients does not significantly differ from that in age- and sex-matched controls from the general population [40,41]. Metabolic syndrome in IBD patients has been linked to higher disease activity, more complications, and increased need for surgical interventions, particularly in CD [25,26]. Although the overall prevalence of metabolic syndrome in IBD appears comparable to that of the general population in matched cohorts, its clinical relevance in IBD may be disproportionate as it is associated with higher disease activity, increased complications, and adverse outcomes [42].

### 3.2. Metabolic Dysfunction-Associated Steatotic Liver Disease

MASLD is a chronic liver condition characterized by the accumulation of fat within hepatocytes, driven by underlying metabolic dysfunction such as obesity, type 2 diabetes mellitus, insulin resistance or other metabolic abnormalities [43]. The term MASLD has recently replaced nonalcoholic fatty liver disease (NAFLD), shifting from a diagnosis of exclusion to a positive definition based on the presence of metabolic risk factors [43]. Most patients previously classified as having NAFLD meet current MASLD criteria; therefore, in this review, NAFLD terminology is used when referring to earlier studies, while MASLD is used according to current nomenclature. Regarding the burden of MASLD in IBD patients, a recent meta-analysis including over 1.5 million individuals found a pooled prevalence of 25.4% (95% CI: 23.1–27.8%) for NAFLD in IBD, with higher rates in adults, males, and European cohorts [20]. Another meta-analysis reported a prevalence of 30.7% (95% CI: 26.5–34.9%) and found that IBD patients have nearly double the risk of NAFLD compared to healthy controls [21]. In addition, IBD has been found to be an independent risk factor for MASLD in lean individuals (21.3% vs. 10%) [44]. Disease activity, disease duration, and specific disease phenotypes, such as penetrating CD, have been reported to be associated with an increased risk of MASLD and its progression, independent of traditional metabolic syndrome features [27]. Clinically, MASLD in IBD is associated with increased comorbidities, impaired outcomes and higher mortality, underscoring the need for early detection and integrated management of both conditions [28].

### 3.3. Obesity

Obesity, a metabolic disorder and a major contributor to both metabolic syndrome and MASLD, has also been widely studied in IBD populations. Large cohort studies indicate that obesity is common among patients with IBD, with reported prevalence rates ranging from 15% to 40%, and an additional 25–40% of patients classified as overweight [29,45,46,47]. Overall, obesity prevalence in IBD appears to be similar to, or slightly lower than, that observed in the general population in matched analyses, although it is increasing in parallel with global obesity trends [29]. Obesity has also been associated with IBD risk in a disease-specific manner. Pooled analyses suggest that obesity confers a 34–42% higher risk of incident CD, with evidence of a dose–response relationship between increasing body mass index and CD risk [22,23]. In contrast, a meta-analysis of prospective studies found that the incidence risk of UC was 21% lower in obese individuals than in normal-weight individuals [48]. Clinically, obesity in IBD is linked to more severe disease activity, higher rates of relapse and worse patient-reported outcomes, including increased anxiety, depression, fatigue and pain [30,31]. Obese IBD patients have been found to experience attenuated response to immunomodulators and biologics, higher rates of perioperative complications, longer hospitalizations, and increased use of steroids and antibiotics [31].

### 3.4. Osteoporosis

Osteoporosis, a metabolic bone disease, is characterized by an imbalance in bone remodeling, specifically between bone formation and bone resorption, leading to decreased bone mass and deterioration of bone microarchitecture, which increases fracture risk [49]. Osteoporosis demonstrated to be a frequent and clinically significant comorbidity in patients with IBD. The prevalence of osteoporosis in patients with IBD, including both CD and UC, has been reported to be higher than that observed in the general population. According to a recent-meta-analysis involving 417,298 patients with IBD, the overall pooled prevalence of osteoporosis in IBD patients was 12.2%. The risk was increased for both disease types, with CD showing a slightly higher prevalence (14.9%) compared to UC (11.4%), though this difference is not statistically significant [24]. Furthermore, osteopenia may affect up to 40% of IBD patients, and the risk of fragility fracture, especially vertebral fracture, appears to be elevated, with a relative risk of 1.38 for any fracture and 2.26 for vertebral fractures compared to non-IBD individuals [32]. The incidence of osteoporosis and fractures has been demonstrated to increase with age, corticosteroid exposure, low body mass index and disease activity, but is not strongly influenced by sex or IBD subtype [33,34].

### 3.5. Diabetes Type 2

Type 2 diabetes affects an estimated 11–14% of the global adult population [50]. A bidirectional association between type 2 diabetes and IBD has been explored in large population-based cohort studies, with additional support from Mendelian randomization analyses. In a UK Biobank cohort including more than 440,000 individuals, patients with IBD were reported to have an increased risk of incident type 2 diabetes (HR: 1.44, 95% CI: 1.31–1.59), with higher risk estimates observed in CD (HR: 1.62, 95% CI: 1.39–1.89) compared with ulcerative colitis (HR: 1.41, 95% CI: 1.13–1.76). Conversely, individuals with type 2 diabetes were reported to have an increased risk of developing IBD (HR: 1.40, 95% CI: 1.15–1.69), with similar risk estimates for UC and CD. In these analyses, the association appeared to be stronger with greater disease severity and was observed across age, sex and BMI strata [51]. In a nationwide Danish cohort including more than six million individuals, patients with IBD were reported to have a 54% higher incidence of type 2 diabetes compared with the general population. The highest relative increase was observed during the first year following IBD diagnosis, while an increased incidence appeared to persist for more than 20 years thereafter [52].

Mendelian randomization studies reveal that the bidirectional association between IBD and type 2 diabetes is not supported as a strong causal relationship. Most recent analyses using large-scale genome-wide association data in European populations show that genetic liability to IBD does not significantly increase the risk of type 2 diabetes, and genetic predisposition to type 2 diabetes does not significantly increase the risk of IBD [53,54]. Overall, these findings suggest that observed epidemiological associations are more likely driven by shared risk factors and comorbidities rather than direct genetic causality [55].

Comorbid diabetes in IBD is associated with worse disease outcomes, including higher rates of IBD-related hospitalizations, disease flares, complications, infections and increased health care utilization and costs [35,36,37]. Quality of life is lower in patients with both conditions, and diabetes may exacerbate IBD severity, as reflected by elevated inflammatory biomarkers and more frequent use of supportive therapies [37]. On the other hand, the use of corticosteroids for IBD may further complicate glycemic control and increase diabetes risk [38].

## 4. Pathophysiological Interactions Between Metabolic Disorders and Inflammatory Bowel Diseases

**Figure 1:**
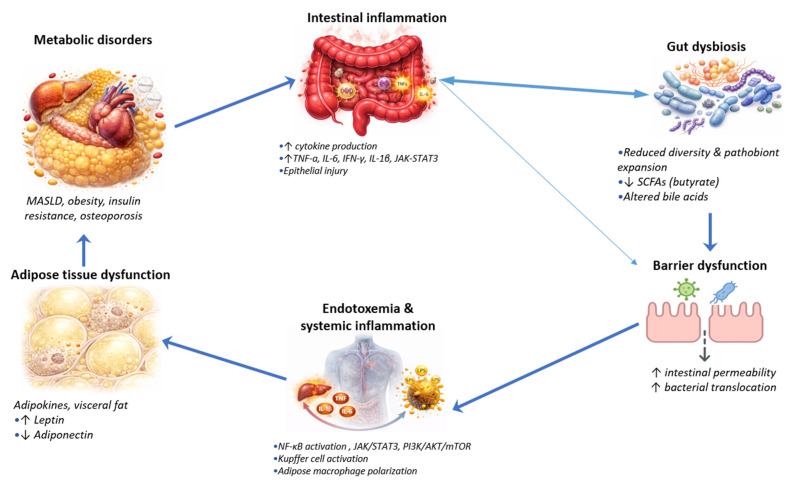
**The vicious cycle linking inflammatory bowel diseases and metabolic disorder**. Intestinal inflammation promotes alterations in the gut microbiota, characterized by reduced diversity and expansion of pathobionts. Dysbiosis contributes to epithelial barrier dysfunction, leading to increased intestinal permeability and bacterial translocation. The passage of microbial products such as lipopolysaccharide into the circulation drives endotoxemia and systemic inflammation, with enhanced cytokine production and hepatic immune activation. Systemic inflammatory signaling promotes adipose tissue dysfunction, adipokine release, and visceral fat expansion, which in turn aggravate insulin resistance and metabolic comorbidities including MASLD, obesity, and osteoporosis. These metabolic disturbances further amplify intestinal inflammation, perpetuating a self-reinforcing pathogenic loop.

Metabolic disorders and inflammatory bowel diseases are connected through several interacting mechanisms involving the intestinal barrier, the gut microbiota, adipose tissue, and immune–metabolic pathways. Rather than representing parallel conditions, these diseases interact at multiple biological levels. Importantly these mechanisms are not uniformly expressed across IBD phenotypes. CD and UC have demonstrated differential metabolic signatures and microbiome derived metabolites, which may contribute to heterogenous susceptibility to metabolic comorbidities. Together, these mechanisms may form a vicious cycle (Figure 1), in which intestinal inflammation and metabolic dysfunction reinforce each other.

### 4.1. Intestinal Barrier Dysfunction

Intestinal barrier dysfunction is increasingly recognized as a key pathophysiological feature linking metabolic disorders and IBDs, largely through increased intestinal permeability, often referred to as “leaky gut”. The intestinal barrier comprises epithelial cells, tight junction proteins, the mucus layer, and immune components, which together regulate the selective absorption of nutrients while limiting the translocation of bacteria, toxins and antigens from the gut lumen into the systemic circulation [56,57]. Disruption of this barrier, as observed in both IBDs and metabolic disorders, may permit the passage of microbial products such as lipopolysaccharide (LPS) and other luminal antigens into the submucosa and bloodstream, and has been associated with local and systemic immune activation. In IBDs, this process may contribute to chronic mucosal inflammation and is influenced by genetic susceptibility (e.g., variants in NOD2 and MUC2) and intestinal dysbiosis, both of which can further compromise barrier integrity and sustain inflammatory responses [56,58]. In metabolic disorders, increased intestinal permeability may similarly facilitates the translocation of microbial components, particularly LPS, into the systemic circulation, where they induce chronic low-grade inflammation [59]. Circulating LPS can activate Toll-like receptor 4 (TLR4) on immune cells and has been shown to trigger the release of pro-inflammatory cytokines such as TNF-α, IL-6, and IL-1β. In adipose tissue, inflammation is linked to alterations in adipokine secretion and lipid metabolism, which may contribute to obesity [60,61,62]. Similarly in the liver, LPS and other microbial products activate Kupffer cells and hepatic stellate cells, driving inflammatory and fibrotic processes that underlie MASLD. This gut-derived inflammatory cascade may contribute to a self-perpetuating cycle, although direct causal pathways in humans remain incompletely defined [28,63].

### 4.2. Gut Microbiota Dysbiosis

Gut microbiota dysbiosis has been proposed to contribute to the pathophysiological links between IBDs and metabolic disorders by altering the balance between beneficial and potentially harmful microbial species, leading to altered production of key metabolites, impaired intestinal barrier function and chronic immune activation [64]. In IBD, dysbiosis is characterized by a reduction in anti-inflammatory bacteria, such as Faecalibacterium and Roseburia, and an increase in pro-inflammatory species (such as Escherichia coli and AIEC), resulting in decreased production of short-chain fatty acids (SCFAs) and secondary bile acids which are essential for maintaining gut barrier integrity and immune homeostasis [65,66]. This microbial imbalance impairs the mucosal barrier, allowing translocation of microbial products (e.g., LPS) and metabolites into the circulation, which triggers local and systemic inflammation [67]. In metabolic disorders, similar patterns of dysbiosis are observed, with reduced SCFA-producing bacteria and increased production of dysbiotic metabolites, which promote low-grade systemic inflammation, insulin resistance, and hepatic lipid dysregulation. Metagenome-wide association studies have demonstrated that patients with metabolic syndrome, type 2 diabetes and MASLD exhibit lower microbial diversity and reduced abundance of SCFA producers [68]. In parallel, an increase in bacteria capable of producing dysbiotic metabolites, including trimethylamine (TMA), hydrogen sulfide (H_2_S) and p-cresyl sulfate, has been demonstrated. These changes are associated with low-grade systemic inflammation, insulin resistance and hepatic lipid dysregulation [69,70]. These shared dysbiotic signatures and metabolite alterations support the concept of a bidirectional association between IBDs and metabolic disorders; however, the current evidence is largely derived from observational and experimental studies, and direct causal relationships in humans have not been firmly established.

It should be acknowledged that microbiome findings in IBDs and metabolic disorders show substantial heterogeneity. Differences in sequencing methodologies (16S rRNA versus shotgun metagenomics), bioinformatic pipelines, and sample handling can significantly influence taxonomic resolution and diversity estimates [71,72]. In addition, microbiota composition is strongly affected by diet, medication exposure (including antibiotics, corticosteroids, biologics, and immunomodulators), BMI, and disease activity, which are not uniformly controlled across studies [72,73]. These factors limit comparability and reproducibility, and therefore reported microbial signatures should be interpreted with caution.

### 4.3. Adipose Tissue Dysfunction and Immunometabolism

Adipose tissue dysfunction is characterised by chronic low-grade inflammation, immune-cell infiltration, and dysregulated adipokine secretion. Together, these alterations drive systemic metabolic disturbance and sustained immune activation [74]. In this state, adipose tissue functions as an immunometabolic organ, with increased production of pro-inflammatory cytokines such as TNF-α and IL-6, alongside elevated levels of adipokines including leptin and resistin, while the anti-inflammatory adipokine adiponectin is reduced. This imbalance has been associated with the development of insulin resistance, type 2 diabetes, and atherosclerosis [75].

Immunometabolism has emerged as a central mechanism governing both innate and adaptive immune regulation, highlighting the inseparable relationship between cellular metabolic pathways and immune function [76]. In both IBDs and metabolic disorders, adipose tissue dysfunction reshapes immune-cell responses and sustains chronic inflammation, thereby perpetuating disease activity and metabolic derangements [75]. Gut dysbiosis, a common feature of IBD and obesity, further fuels this vicious cycle by increasing intestinal permeability and systemic exposure to microbial products. These signals activate adipose depots and circulating immune cells, maintaining a state of persistent meta-inflammation [17].

In IBDs, particularly CD, mesenteric adipose tissue—commonly referred to as “creeping fat”—has been shown to produce inflammatory mediators that may amplify intestinal inflammation and fibrosis [77]. Compared with healthy adipose tissue, creeping fat demonstrates pronounced immune-cell infiltration, altered adipokine profiles, and enhanced secretion of pro-inflammatory cytokines, underscoring its role as an active immunological organ rather than a passive energy store. Beyond local effects, this dysfunctional tissue can influence systemic immunity, promoting fibrosis, angiogenesis, and wider metabolic perturbations [78,79]. Clinical and experimental studies further associate these adipose tissue alterations with changes in body composition, increased disease severity, and unfavourable outcomes, with leptin, adiponectin, and resistin acting as central mediators at the interface between inflammation and metabolism [11,80].

### 4.4. Bone Metabolism and Cytokine-Driven Skeletal Remodeling

Bone loss in IBD appears to result from a complex interplay of cytokine-driven bone resorption, impaired bone formation, and nutritional deficiencies, extending beyond corticosteroid exposure alone.

Pro-inflammatory cytokines—including TNF-α, IL-1β, IL-6, IL-17, and interferon-γ—have been implicated in promoting osteoclast differentiation and activity while inhibiting osteoblast function [81]. TNF-α, a central cytokine in IBD, induces osteoclast-mediated bone erosion in experimental models. A subset of CD4+ T cells producing IL-17 and TNF-α (Th17/TNF-α+ cells) has been identified as highly osteoclastogenic; during chronic inflammation, these cells may migrate to the bone marrow, promote inflammatory monocyte recruitment, and thereby link intestinal inflammation to enhanced bone resorption [82].

Dysregulation of the RANKL/osteoprotegerin (OPG) axis represents a key mechanism. RANKL stimulates osteoclastogenesis via RANK signaling, whereas OPG acts as a decoy receptor. Although circulating OPG levels are elevated in IBD, the RANKL/OPG ratio may remain increased, particularly in CD, and has been associated with reduced bone mineral density [83,84]. Increased osteocyte expression of RANKL and sclerostin, together with local production of TNF-α and IL-6, further suggests a shift toward enhanced bone resorption and suppressed bone formation. Experimental data also indicate that intestinal epithelial NF-κB activation may contribute to systemic osteoclastogenic signaling, although human data remain limited [84,85].

Malabsorption and nutritional deficiencies substantially contribute to IBD-associated osteoporosis, especially in CD with ileal involvement. Vitamin D deficiency is highly prevalent and may promote bone loss through reduced calcium absorption, impaired osteoblast function, and loss of its immunomodulatory effects [86]. Vitamin D/VDR signaling influences epithelial barrier integrity, antimicrobial responses, and T-cell polarization, suggesting that deficiency may indirectly exacerbate inflammation-driven bone resorption [87]. Moreover, calcium malabsorption in IBD patients, particularly after small bowel inflammation or resection, may directly impair bone mineralization [34].

Overall, IBD-associated osteoporosis likely reflects an integrated immunometabolic process in which chronic intestinal inflammation promotes systemic cytokine signaling and RANKL/OPG imbalance, while concurrent nutritional deficiencies may reduce the skeleton’s capacity to maintain bone homeostasis.

### 4.5. Dietary and Environmental Factors

Dietary and environmental factors have been suggested as potential modulators of immunometabolic interactions, acting via changes in microbiota composition, epithelial barrier function, and immune and metabolic signaling networks [88]. Diet characterized by high intake of saturated fats, processed meats, and refined sugars have been associated with increased risk and greater disease severity in both IBDs and metabolic conditions, including obesity, type 2 diabetes, and metabolic syndrome. Such exposures are linked to reduced microbial diversity, expansion of pro-inflammatory taxa, and impairment of barrier function, changes that may facilitate enhanced immune activation and the maintenance of chronic low-grade inflammation [89,90]. Conversely, diets rich in fiber, fruits, vegetables, and whole grains, such as the Mediterranean diet, have been linked to lower inflammation burden, improved metabolic profiles, and greater microbial diversity [91,92]. In IBD patients, higher fiber intake has been associated with a lower incidence of CD and has a positive influence on gut barrier function and microbial composition, which also benefits metabolic health. Poor dietary quality and low diversity are common among IBD patients and are associated with an increased risk of metabolic complications [93,94].

Environmental factors such as smoking tobacco, living in an urban area, exposure to antibiotics, vitamin D deficiency, physical inactivity, exposure to environmental pollutants (including air pollution, pesticides and heavy metals) and psychosocial stress may alter the gut microbiome and immune responses, contributing to both IBDs and metabolic disorders by promoting dysbiosis, increased intestinal permeability, and chronic inflammation [95]. Tobacco smoking has been associated with shifts in gut microbiota composition, increasing pro-inflammatory taxa (e.g., Proteobacteria) and reducing beneficial short-chain fatty acid producers, which may impair mucosal immunity and barrier function, thereby exacerbating CD and metabolic dysfunction [96,97]. Urban living has been associated with higher exposure to pollutants and reduced microbial diversity, which correlates with increased risk of both IBDs and metabolic disorders through mechanisms involving chronic low-grade inflammation and immune dysregulation [98,99]. Antibiotic exposure may disrupt microbial diversity, favoring pathogenic bacteria and reducing protective species, leading to dysbiosis and heightened immune activation, which is implicated in both IBD and metabolic syndrome [100,101].

Physical activity has been inversely associated with the development and progression of both IBD and metabolic disorders. The protective effect appears to be mediated through multiple interacting mechanisms, including attenuation of systemic inflammation, favorable modulation of the gut microbiota, improvement of metabolic parameters, and enhancement of intestinal barrier integrity [102]. In IBD, moderate-to-vigorous physical activity has been linked to reduced incident disease risk, particularly for CD, and has been associated with lower rates of disease flare, improved quality of life, and decreased mortality [103]. Studies have suggested that exercise may induce favourable circulating metabolite profiles, increase microbial diversity, and promote secretion of anti-inflammatory myokines, which collectively attenuate intestinal inflammation and enhance barrier function [104,105,106]. On the other hand, physical inactivity, conversely, has been linked to higher IBD risk and exacerbation of coexisting metabolic disorders such as metabolic syndrome and steatotic liver disease [107,108]. Regarding metabolic disorders, regular physical activity substantially decreases the risk of metabolic syndrome, type 2 diabetes, and cardiovascular disease. These benefits are primarily attributed to enhanced insulin sensitivity, improved lipid metabolism, reduction in visceral adiposity, and overall suppression of low-grade inflammatory pathways [109].

### 4.6. Inflammatory Biomarkers and Clinical Correlations

The interaction between IBDs and metabolic disorders is mediated through a shared immunometabolic axis. Elevated circulating levels of C-reactive protein (CRP) and interleukin-6 (IL-6) have been correlated with increased disease activity in both CD and UC and reflect systemic inflammatory burden [110]. Higher CRP and IL-6 levels have also been associated with insulin resistance, dyslipidemia, and adverse cardiometabolic profiles, suggesting that persistent inflammation may contribute to metabolic dysregulation [111,112].

Adipocytokine imbalance represents a key mechanistic link. Increased leptin and reduced adiponectin levels have been observed in active IBDs and are associated with greater inflammatory activity and metabolic impairment [113]. Leptin exerts pro-inflammatory effects by promoting immune cell activation and cytokine production, whereas adiponectin, which generally has anti-inflammatory and insulin-sensitizing properties, is reduced during active disease. This dysregulation has been demonstrated to be evident in mesenteric adipose tissue (“creeping fat”) in CD, which actively secretes cytokines and adipokines that may amplify both intestinal and systemic inflammation [114,115,116].

### 4.7. Differential Metabolic Expression in IBD: A Complex Immunometabolic Framework

Accumulating evidence have demonstrated that CD and UC exhibit distinct metabolic profiles, both at the level of circulating metabolites and tissue-specific metabolic pathways. Metabolomic studies consistently show that CD is characterized by more pronounced alterations in lipid, amino acid, and energy metabolism compared to ulcerative colitis, which tends to display milder metabolic changes and distinct perturbations in sphingolipid and bile acid metabolism [117,118].

The gut microbiome and its metabolic activity differ substantially between CD and UC. In CD, a pronounced reduction in overall microbial diversity is consistently observed, particularly involving depletion of Firmicutes and Bacteroidetes, alongside enrichment of Proteobacteria and Actinobacteria, including adherent-invasive Escherichia coli. Notably, butyrate-producing taxa such as Faecalibacterium prausnitzii and Roseburia spp. are significantly reduced. This depletion impairs short-chain fatty acid production, compromises epithelial barrier integrity, and attenuates anti-inflammatory signaling [119,120,121]. CD-associated dysbiosis is further linked to alterations in amino acid metabolism—particularly branched-chain amino acids—and disrupted bile acid composition, promoting pro-inflammatory cytokine activation, metabolic stress, and impaired host energy homeostasis [122]. In contrast, UC typically demonstrates a less marked reduction in global microbial diversity, although depletion of beneficial commensals, including Faecalibacterium prausnitzii and Akkermansia muciniphila, remains evident. Metabolic perturbations in UC more prominently involve nitrogen and butanoate metabolism, as well as specific alterations in tryptophan and carnitine pathways. These changes may influence mucosal immune regulation and epithelial turnover rather than systemic energy metabolism to the same extent as in CD [123,124].

These subtype-specific microbial–metabolic alterations likely contribute to distinct inflammatory signaling and systemic metabolic consequences across IBD phenotypes.

Collectively, these mechanisms should not be viewed as isolated processes but rather as components of an interconnected immunometabolic axis. Intestinal barrier dysfunction may facilitate microbial translocation, which has been closely linked to dysbiosis and altered metabolite production. These signals may interact with adipose tissue depots and systemic immune pathways, potentially contributing to chronic low-grade inflammation and metabolic dysregulation [59,125]. In turn, metabolic inflammation may further influence barrier integrity and microbial balance, suggesting the presence of a self-reinforcing network rather than parallel mechanisms [17]. This integrated framework may help explain the bidirectional amplification between intestinal inflammation and metabolic dysfunction observed in IBD.

## 5. Treatment Interactions Between IBDs and Metabolic Disorders

The coexistence of IBDs and metabolic disorders presents important therapeutic considerations. Treatments targeting intestinal inflammation may also influence systemic metabolic pathways, affecting glucose regulation, lipid profiles and bone metabolism (Table 3). At the same time, metabolic comorbidities can modify drug response, safety, and long-term outcomes. Given the increasing prevalence of obesity, metabolic syndrome, diabetes, and steatotic liver disease among patients with IBD, awareness of these bidirectional interactions is essential.

### 5.1. Corticosteroids-Related Metabolic Effects

Despite the development of newer advanced therapies over recent years, corticosteroids continue to be widely used in clinical practice, particularly for the induction of remission and the management of acute disease flares. Their use has long been associated with a broad spectrum of metabolic disturbances, including hyperglycemia, insulin resistance, hypertension, weight gain—particularly central or visceral adiposity—and dyslipidemia. These adverse effects are generally dose- and duration-dependent; however, accumulating evidence suggests that even low-dose, chronic exposure may confer clinically meaningful risk [126]. Mechanistically, corticosteroids may increase hepatic gluconeogenesis and decrease peripheral glucose uptake, leading to hyperglycemia and insulin resistance. They also promote adipocyte differentiation and visceral fat accumulation, resulting in central obesity. Corticosteroid use has been associated with alteration of lipid metabolism, causing dyslipidemia. Moreover, sodium retention and heightened vascular reactivity may lead to elevated blood pressure. Long-term therapy also negatively affects bone metabolism and increases the likelihood of osteoporosis [127,128].

### 5.2. Biological Therapies-Related Metabolic Effects

Biologic therapies used in IBD, including anti-TNF agents (infliximab, adalimumab, certolizumab pegol, golimumab), anti-integrin agents (vedolizumab, natalizumab), and anti-IL-12/23 therapy (ustekinumab), appear to exert differential effects on metabolic pathways. Anti-TNF therapy has been associated with modest weight gain and with a higher likelihood of overweight or obesity, particularly when combined with immunomodulators. This increase in body weight is commonly interpreted as a consequence of improved inflammatory control, reversal of catabolism, and recovery of nutritional status [129,130]. In addition to these remission-related mechanisms, TNF inhibition may also exert direct metabolic effects through modulation of adipokine secretion and insulin signaling pathways. Nevertheless, a subset of patients may develop pronounced increases in total body weight and visceral adiposity. Beyond anthropometric changes, anti-TNF agents suppress adipose tissue inflammation and may improve adipose tissue architecture, especially in CD, although they do not reliably reduce visceral fat mass [131]. Importantly, several studies indicate that anti-TNF treatment may enhance insulin sensitivity in non-obese, non-diabetic patients with IBD. Improvements have been documented through reductions in HOMA-IR, fasting insulin concentrations, and c-peptide levels, often occurring without major short-term changes in body weight or BMI. These findings suggest that attenuation of systemic inflammation can beneficially modulate glucose metabolism independently of weight variation [132,133].

Vedolizumab, an anti-integrin agent, has also been associated with increased odds of overweight and obesity in IBD patients. The mechanism is less well characterized, but weight gain appears to be a class effect among biologics, possibly related to disease remission and improved nutritional status [130].

Current evidence does not support a clinically meaningful association between anti–IL-23 therapies—including ustekinumab, risankizumab, guselkumab, and mirikizumab—and weight gain or worsening insulin sensitivity in patients with IBD. Across randomized controlled trials and network meta-analyses, rates of metabolic adverse events are low and generally comparable to placebo or other biologic therapies during approximately one year of follow-up [134,135]. For ustekinumab, extended data from psoriasis and psoriatic arthritis cohorts have not demonstrated increased risk of weight gain or incident diabetes, while abnormalities in non-fasting glucose have been infrequent and not clearly drug related [136]. Similarly, studies of risankizumab, guselkumab, and mirikizumab—including populations with overweight or obesity—indicate preserved therapeutic efficacy without consistent signals for treatment-emergent changes in body weight or metabolic parameters through 52 weeks [137].

Overall, available evidence suggests that weight gain during biologic therapy may reflect a combination of inflammatory control and potential direct metabolic effects, with their relative contribution likely varying according to baseline adiposity and individual patient characteristics.

### 5.3. Janus Kinase Inhibitors and Their Metabolic Effects

Recently, Janus kinase (JAK) inhibitors represent an important therapeutic option in management of IBD. Regarding their metabolic effects, they appear to have a neutral effect on body weight and insulin sensitivity. Data from randomized trials and real-world cohorts evaluating tofacitinib, upadacitinib, and filgotinib do not show clinically meaningful increases in body weight, visceral adiposity, or deterioration of glycemic parameters in patients with UC or CD. Moreover, treatment response seems independent of baseline body mass index, suggesting that obesity does not substantially modify efficacy [138,139,140]. JAK inhibitors have been linked to reversible, dose-dependent increases in serum lipid levels. This effect has been consistently reported with tofacitinib, upadacitinib and filgotinib, typically emerging within weeks of treatment initiation and affecting both LDL and HDL cholesterol levels. The magnitude of the change is usually modest, with lipid concentrations returning towards baseline after a reduction in dosage or discontinuation of treatment [141,142]. Moreover, the LDL-C/HDL-C ratio does not significantly change with JAK inhibitor therapy, as both fractions increase proportionally [143]. Regarding cardiovascular safety of Janus kinase inhibitors in IBD, a systematic review and network meta-analysis of 26 randomized controlled trials including 10,537 patients found no significant increase in the risk of major adverse cardiovascular events, venous thromboembolism, or overall cardiovascular events associated with Janus kinase inhibitor therapy [142]. Furthermore, a recent multicenter cohort study comparing JAK inhibitors with anti–TNF therapy in 17,884 patients with IBD demonstrated no significant difference in the incidence of major adverse cardiovascular events (aHR 1.08, 95% CI 0.87–1.33) or venous thromboembolism (aHR 1.06, 95% CI 0.84–1.36) at 12 months of follow-up, including among patients aged 65 years or older [144]. However, individuals with pre-existing cardiovascular risk factors may benefit from closer surveillance and routine lipid monitoring is recommended during therapy, and management of dyslipidemia should follow standard cardiovascular risk guidelines [145].

### 5.4. Anti-Diabetic Agents and IBDs

The potential effects of antidiabetic agents in IBDs derive from a combination of preclinical studies, observational data, and limited clinical trial evidence, and therefore should be interpreted according to the level of available evidence.

Glucagon-like peptide-1 receptor agonists (GLP-1 RAs) have been reported to be safe and well tolerated in patients with IBD, and have been associated with significant weight loss, improved metabolic parameters, and a reduction in IBD-related hospitalizations and surgery, particularly in patients with obesity [146]. In preclinical models, GLP-1 RAs have been demonstrated to have anti-inflammatory effects and have been associated with reductions in C-reactive protein, though IBD-specific trials are needed to confirm disease-modifying effects [147]. The metabolic benefits in patients with IBD, including weight loss and improved glycemic control, are robust and comparable to those seen in non-IBD populations [148].

The administration of metformin has been associated with a reduction in the activity and severity of IBD by activating AMP-activated protein kinase, suppressing pro-inflammatory cytokines (IL-1β, IL-6 and TNF-α), improving gut barrier integrity and modulating the gut microbiota. Observational clinical studies suggest possible associations with reduced disease activity and lower steroid requirements, although causal relationships have not been established [149,150,151]. As peroxisome proliferator-activated receptor gamma (PPARγ) agonists, thiazolidinediones have been shown to attenuate colitis in animal models and may reduce the risk of significant flare-ups requiring steroid treatment in patients with ulcerative colitis. However, the clinical benefit of thiazolidinediones over other oral antidiabetic agents is not clearly established. Thiazolidinediones have also been demonstrated to downregulate proinflammatory cytokine signalling, particularly the IL-6 and STAT3 pathways [152,153].

SGLT2 inhibitors reduce pro-inflammatory M1 macrophage polarization and promote anti-inflammatory M2 phenotypes by inhibiting key signaling pathways like NF-κB, PI3K/AKT/mTORC1, and JAK/STAT [154]. They may also decrease NLRP3 inflammasome activation, resulting in lower secretion of inflammatory markers such as IL-1β, IL-6, and TNF-α, which reduces systemic and tissue-specific inflammation, regardless of glycemic control [155]. A nationwide cohort study found that SGLT2 inhibitor use significantly lowers the risk of developing IBD compared to DPP-4 inhibitors, with an adjusted hazard ratio of 0.39 (95% CI 0.24–0.65). This gut-protective effect is likely due to the suppression of macrophage-driven inflammation and inflammasome activity in the intestinal mucosa [156]. It should be noted that, in a meta-analysis of 198,404 patients, there was no evidence that DPP-4 inhibitors increase the risk of developing IBD [157]. Nevertheless, these findings are hypothesis-generating, and further studies are required.

### 5.5. Antilipidemic Agents and IBDs

Antilipidemic agents have been found to have immunomodulatory and anti-inflammatory properties that may affect the risk of and progression of IBDs, but the impact varies depending on the drug class. Statins are linked to a reduced risk of developing IBDs, especially CD, and may help lower disease activity and improve outcomes in patients with IBD. Their protective effect is most significant in older adults and is consistent across various statin types [158,159]. In patients with established IBD, statins are associated with a milder disease course, leading to fewer surgeries, hospitalizations, and flare-ups, particularly in UC [160,161]. These benefits are believed to result from statins’ immunomodulatory effects, such as inhibiting T-cell activation and reducing inflammatory cytokines [162]. While preclinical studies support the anti-inflammatory properties of statins, further research is needed to understand their molecular mechanisms and optimal use in IBD. Overall, statins may serve as adjunctive therapy for managing cardiovascular risk in IBD patients, with potential added benefits for disease activity [163].

Regarding the other antilipidemic agents, genetic and Mendelian randomization studies suggest that inhibiting lipid targets like PCSK9, NPC1L1, ANGPTL3, and APOC3 may increase the risk of IBD [164,165]. Conversely, enhancing LDL receptor (LDLR) and lipoprotein lipase (LPL) activity is linked to a reduced risk of IBD, partly through changes in gut microbiota and inflammatory cytokines. Ezetimibe, which inhibits NPC1L1, may raise the risk of ulcerative colitis, while CETP inhibitors that increase HDL cholesterol (HDL-C) show promise in reducing disease severity and inflammation in colitis models [165]. Combination therapies with statins and ezetimibe may provide anti-inflammatory benefits mainly due to statins. Other lipid-lowering agents like bempedoic acid and omega-3 fatty acids can reduce inflammatory markers, but their effects on IBD outcomes are less clear [166,167].

## 6. Screening Strategies and Management

Considering the substantial burden and prognostic implications of metabolic comorbidities in IBD, early identification through systematic screening is essential in contemporary clinical practice. Screening strategies for metabolic disorders in patients with IBD should include regular assessments of BMI, waist circumference, blood pressure, fasting glucose levels, and lipid profiles. Non-invasive hepatic steatosis scores, such as the MAFLD-S, Fatty Liver Index, Hepatic Steatosis Index, and Clinical Prediction Tool for NAFLD in CD (CPN-CD), have shown high accuracy in identifying MASLD in IBD patients [168]. These tools can be used to select individuals for further hepatic evaluation, such as transient elastography with controlled attenuation parameter (CAP), to detect steatosis and fibrosis [169,170]. Additionally, lifestyle factors, including physical inactivity and poor dietary habits, further elevate metabolic risks and should be evaluated during the screening process. Routine laboratory screening for micronutrient deficiencies—such as iron, vitamin D, vitamin B12, folate, and zinc—is also advisable, as these deficiencies are common in IBD patients and can contribute to metabolic dysfunction [171,172].

Once metabolic risk or established metabolic disease is identified, management should follow an integrated and multidisciplinary strategy aimed at reducing systemic inflammation, preventing end-organ damage, and optimizing IBD outcomes. Importantly, therapeutic plans should account for the bidirectional interaction between intestinal inflammation and metabolic dysfunction, recognizing that effective control of one domain may positively influence the other. Dietary management is essential for IBD patients with metabolic disorders. The Mediterranean diet is recommended unless contraindicated, as it promotes better metabolic health, reduces cardiovascular risk, and may positively impact IBD activity and quality of life [173,174]. This diet highlights fresh fruits, vegetables, monounsaturated fats, complex carbohydrates, and lean proteins while reducing ultra-processed foods, added sugars, and salt. Structured diets, such as the specific carbohydrate diet, CD exclusion diet, and low-FODMAP diet, can help control symptoms and lower metabolic risk, especially in patients with active disease or gastrointestinal symptoms. Careful monitoring for nutritional deficiencies, particularly in iron, vitamin D, B12, folate, and zinc, is necessary with restrictive diets, which should be personalized based on disease activity and individual preferences [175]. Lifestyle modifications are crucial for the effective management of IBDs and metabolic disorders. Regular physical activity, weight management, and minimizing sedentary behavior are vital for improving health outcomes. Obesity and changes in body composition, such as excess visceral fat and reduced muscle mass, can worsen IBD outcomes and increase metabolic risks. Therefore, maintaining a healthy weight and muscle mass is important for disease control and reducing metabolic complications [173]. Multidisciplinary and personalized care is crucial for effective management. Involving dietitians and a diverse healthcare team allows for individualized dietary counseling and the optimization of nutritional status. While existing IBD guidelines provide recommendations for selected metabolic aspects—particularly bone health and corticosteroid-related complications—comprehensive guidance addressing the optimal frequency of screening across the broader spectrum of metabolic comorbidities remains limited. Consequently, clinicians often rely on extrapolation from general population and disease-specific metabolic guidelines. In this context, we propose a pragmatic, evidence-informed framework to support metabolic risk assessment in clinical practice (Table 4). The suggested intervals should be interpreted as flexible guidance and individualized according to disease activity, treatment exposure, and overall cardiometabolic risk profile.

Regular assessments of nutritional status, body composition, and key metabolic parameters are essential for guiding interventions. Point-of-care body composition measurements can help identify and address myopenia and visceral obesity, which are linked to negative health outcomes [171]. However, advanced body composition and imaging-based assessments may not be universally available and can involve additional costs. In routine practice, widely accessible measures such as body mass index, waist circumference, and basic laboratory markers remain the foundation of metabolic risk assessment [176]. More specialized evaluations may be reserved for high-risk patients or centers with appropriate resources.

Finally, persistent intestinal inflammation is a key driver of metabolic derangement. Achieving sustained remission through treat-to-target strategies may improve insulin resistance, mitigate sarcopenia, and reduce cardiovascular risk. Consequently, early escalation to effective steroid-sparing regimens should be considered when appropriate [177,178]. Minimization of corticosteroid exposure is particularly critical, given their well-documented association with hyperglycemia, visceral adiposity, hypertension, dyslipidemia, and bone loss.

## 7. Limitations

This review has several limitations that should be acknowledged. First, the available literature is characterized by substantial heterogeneity in study design, population characteristics, definitions of metabolic comorbidities, and outcome measures. The included evidence comprises population-based cohorts, meta-analyses, and mechanistic investigations, which limits direct comparability.

Second, most epidemiological associations between IBDs and metabolic disorders are derived from observational studies. Although these studies provide important real-world insights, they are inherently susceptible to residual confounding, including age, body mass index, smoking, medication exposure (particularly corticosteroids), and comorbidity burden. Reverse causality cannot be fully excluded in bidirectional associations. In addition, potential publication bias may favor studies reporting positive associations.

Third, although several studies provide stratification by IBD subtype (CD versus UC) and, less frequently, by disease severity, this stratification is often limited and not uniformly detailed across all cohorts. This likely reflects both the intrinsic heterogeneity of IBD phenotypes and differences in study methodology. More granular, prospective studies incorporating standardized severity indices, longitudinal metabolic assessment, and detailed treatment exposure data are needed to clarify differential risks and causal pathways.

Finally, as a narrative review, this manuscript did not include formal risk-of-bias assessment or meta-analytic pooling, which may limit the strength of inferential conclusions. Nevertheless, we prioritized large population-based studies and recent meta-analyses to ensure contemporary and clinically relevant synthesis.

## 8. Conclusions

The growing recognition of the close relationship between metabolic disorders and inflammatory bowel diseases has reshaped our understanding of both conditions. Rather than representing independent comorbidities, metabolic abnormalities and intestinal inflammation share common pathways involving gut barrier dysfunction, microbiota alterations, immune activation, and adipose tissue-driven meta-inflammation. These overlapping mechanisms help explain the increased burden of obesity, metabolic syndrome, steatotic liver disease, osteoporosis, and type 2 diabetes observed in patients with IBD, as well as the impact of these conditions on disease severity, therapeutic response, and long-term outcomes. Importantly, these metabolic comorbidities exert a clinically meaningful impact on disease severity and outcomes, even when their absolute prevalence is not consistently higher than that of the general population.

At the same time, treatments used to control intestinal inflammation may exert important metabolic consequences, both beneficial and adverse, further highlighting the need for integrated care. Early recognition of metabolic risk factors, systematic screening, and multidisciplinary management strategies that incorporate dietary optimization, physical activity, and appropriate pharmacologic interventions are essential. Furthermore, achieving sustained control of inflammation while minimizing steroid exposure remains a central goal. Future research should aim to clarify causal mechanisms, identify predictive biomarkers, and develop targeted interventions that address immunometabolic pathways. A deeper understanding of this bidirectional relationship will support more personalized therapeutic approaches and improve patients’ quality of life.

## Figures and Tables

**Table 1:** Key Epidemiological Studies on Metabolic Comorbidities in IBDs.

Study Design	Sample Size	Country	Study Population	Metabolic Condition	Key Findings	Clinical Correlation	Limitations	Ref. (Year)
Systematic review & meta-analysis of observational studies	2501 IBD patients (11 studies)	Multinational	Adult patients with CD and UC	Metabolic syndrome	Pooled prevalence 19.4% (95% CI 15.1–23.8%); significantly higher in UC vs. CD (38.2% vs. 13.6%; OR 2.11, 95% CI 1.19–3.74)	Older age identified as risk factor; higher prevalence in UC	Moderate heterogeneity (I^2^ = 51.8%); limited non-IBD controls; predominantly cross-sectional data	[19] (2024)
Systematic review & meta-analysis of observational studies	1,532,811 individuals (64 studies)	Multinational	Adult and pediatric patients with CD and UC; geographic variability	Metabolic dysfunction-associated steatotic liver disease	Pooled prevalence 25.4% (95% CI 23.1–27.8%); higher in adults (26%) vs. pediatric (7%); males 32.1% vs. females 22.9%; similar prevalence in CD (22.8%) and UC (21.4%)	Higher prevalence in CD with upper GI involvement and in UC with left-sided colitis; no significant association with IBD medications	High heterogeneity; diagnostic method variability; limited subgroup data	[20] (2026)
Systematic review & meta-analysis of observational studies	14,947 adult IBD patients (44 studies; 18 countries)	Multinational	Adult patients with CD and UC (≥50 participants per study); heterogeneous diagnostic methods; assessed metabolic risk factors	Metabolic dysfunction-associated steatotic liver disease	Pooled prevalence 30.7% (95% CI 26.5–34.9); OR 1.96 vs. healthy controls; no significant difference CD vs. UC (OR 1.16)	Age and BMI independently associated with NAFLD; advanced fibrosis prevalence 13.6%	High heterogeneity; variable diagnostic criteria (imaging, histology, steatosis index); observational data	[21] (2022)
Pooled analysis of 5 prospective cohorts	601,009 adults; 10.1 million person-years; 563 CD and 1047 UC incident cases	Multinational	Adult population (18–98 years) with validated BMI and waist-hip ratio; incident CD and UC confirmed	Obesity	Obesity (BMI ≥ 30) associated with increased CD risk (aHR 1.34, 95% CI 1.05–1.71); each 5 kg/m^2^ BMI increase → 16% higher CD risk; no association with UC	Dose–response relationship observed for CD; association stronger for early-adulthood BMI	Limited generalizability beyond cohort populations	[22] (2022]
Systematic review & dose–response meta-analysis of cohort studies	>1,000,000 participants	Multinational	General adult population from prospective cohort studies; incident CD and UC cases	Obesity (BMI)	Higher BMI associated with increased risk of CD; dose–response relationship observed; no consistent association with UC	Progressive increase in CD risk with rising BMI	Observational cohort data; residual confounding possible	[23] (2019)
Systematic review & meta-analysis of cross-sectional studies	417,298 adult IBD patients (24 studies)	Multinational	Adult patients (≥18 years) with CD and UC; ≥100 patients per study	Osteoporosis	Pooled prevalence 12.2% (95% CI 9.1–15.3); higher risk vs. controls (OR 1.64, 95% CI 1.24–2.16); no significant difference CD vs. UC	Increased fracture risk implication; prevalence not significantly influenced by disease type or sex	Very high heterogeneity (I^2^ = 99.7%); cross-sectional design	[24] (2025)

**Table 2:** Interaction between Metabolic Comorbidities and IBDs: Impact on Disease Course and Outcome.

Metabolic Condition	Evidence Base (Study Type)	Impact on IBD Activity/Severity	Therapeutic Implications	Long-Term Outcomes/Complications	Evidence (Ref.)
Metabolic syndrome	Meta-analysis + cohort studies	Associated with higher disease activity and increased complication burden; more pronounced signals in CD	Need for dose optimization and steroid-sparing strategies.	Linked to increased need for surgical interventions and adverse clinical outcomes, particularly in CD	[19,25,26]
Metabolic dysfunction-associated steatotic liver disease	Μeta-analyses + cohorts	Associated with higher IBD activity, longer disease duration, and penetrating CD phenotype	Routine liver assessment; optimize metabolic risk factors	Increased comorbidities, worse clinical outcomes, higher mortality; risk of fibrosis progression	[21,27,28]
Obesity	Μeta-analyses + cohorts	Linked to more severe clinical course, higher relapse frequency, and worse patient-reported symptom burden	May attenuate response to biologics; consider weight-adjusted dosing and early optimization; increased need for steroid-sparing strategies; perioperative risk stratification	Higher perioperative complications, longer hospitalizations, increased steroid/antibiotic use	[29,30,31]
Osteoporosis	Meta-analyses + cohorts	Associated with active disease, corticosteroid exposure and low BMI; reflects cumulative inflammatory burden	Minimize corticosteroid exposure; incorporate bone-protective strategies in long-term IBD management	Increased fragility and vertebral fracture risk; age-related progression	[24,32,33,34]
Type 2 diabetes	Large prospective cohorts + nationwide cohorts + systematic reviews	Associated with higher flare rates, hospitalizations, infections and inflammatory burden	Prefer steroid-sparing regimens	Increased complications, healthcare utilization and reduced quality of life	[35,36,37,38]

**Table 3:** Metabolic effects of IBD therapies.

Therapy Class	Body Weight/Adiposity	Glucose Metabolism & Insulin Sensitivity	Lipid Profile	Bone Metabolism
Corticosteroids	Weight gain Increased visceral fat	Hyperglycemia Elevated insulin resistance Elevated risk of diabetes	↑ triglycerides ↑ LDL	Bone loss Increased risk for osteoporosis & fractures
Anti-TNF agents	Mild–moderate weight gain (often with remission)	May improve insulin sensitivity Decreased HOMA-IR reported	Neutral	Neutral/possible indirect benefit via inflammation control
Anti-integrin (vedolizumab)	Possible weight gain (less defined)	Neutral	Neutral	Neutral
Anti-IL-12/23 or IL-23 inhibitors	Neutral	Neutral	Neutral	Neutral
JAK inhibitors	Neutral	Neutral	Reversible ↑ LDL & HDL	Neutral

IL: Interleukin; LDL: Low-density lipoprotein; HDL: High-density lipoprotein; HOMA-IR: Homeostasis Model Assessment—Insulin Resistance.

**Table 4:** Suggested screening strategy for metabolic comorbidities in patients with IBD.

Screening Component	Suggested Frequency	Practical Notes
History & lifestyle assessment	At diagnosis and at least annually	Smoking status, alcohol intake, habitual diet quality, physical activity, sedentary behavior. Reassess after major therapeutic changes or weight variation.
Anthropometrics	At diagnosis and every visit	Body weight, height, BMI, and waist circumference. Consider sarcopenic obesity even with normal BMI.
Blood pressure	At diagnosis and annually (or every visit in high-risk patients)	Screen for hypertension, particularly in obesity, diabetes, and corticosteroid exposure.
Glucose metabolism	At diagnosis and annually	Fasting plasma glucose and/or HbA1c. Monitor more frequently during and after corticosteroid treatment.
Lipid profile	At diagnosis and annually, before and 4–12 weeks after initiation of JAK inhibitors	Total cholesterol, LDL-C, HDL-C, triglycerides. JAK inhibitors are associated with dose-dependent, usually reversible lipid increases; treat according to standard cardiovascular risk guidelines.
Liver function tests	At diagnosis and annually, or as clinically indicated	ALT, AST, ALP, GGT, bilirubin. Abnormalities should prompt evaluation for MASLD, viral hepatitis, biliary disease, and treatment-related hepatotoxicity.
Non-invasive liver assessment	At diagnosis in at-risk patients; otherwise as indicated	Use steatosis indices (e.g., FLI, HSI) and/or transient elastography (CAP) to identify steatosis and fibrosis.
Body composition	As indicated	Waist-to-hip ratio or imaging/DXA when sarcopenia, visceral obesity, or discordance with BMI is suspected.
Micronutrient screening	At diagnosis and periodically according to phenotype	Iron studies, vitamin D, B12, folate, and zinc. More frequent in small-bowel disease, prior resections, or restrictive diets.
Metabolic syndrome assessment	At diagnosis and annually	Apply validated criteria (e.g., NCEP ATP III, IDF). Guides cardiovascular prevention strategies.

The suggested screening intervals are not derived from a single disease-specific guideline for IBD. They represent a synthesis of available evidence from IBD, endocrinology, hepatology and cardiovascular guidelines, observational data, and expert clinical judgment. Screening frequency should be individualized according to patient age, disease activity, corticosteroid exposure, metabolic risk profile, and local resource availability.

## Data Availability

No new data were created or analyzed in this study.
